# The Effects of Random Porosities in Resonant Frequencies of Graphene Based on the Monte Carlo Stochastic Finite Element Model

**DOI:** 10.3390/ijms22094814

**Published:** 2021-05-01

**Authors:** Liu Chu, Jiajia Shi, Yue Yu, Eduardo Souza De Cursi

**Affiliations:** 1 School of Transportation and Civil Engineering, Nantong University, Nantong 226019, China; chuliu@ntu.edu.cn; 2 School of Scienece, Nantong University, Nantong 226019, China; yu.y@ntu.edu.cn; 3 Département Mécanique, Institut National des Sciences Appliquées de Rouen, 76801 Rouen, France; souza@insa-rouen.fr

**Keywords:** random porosities, resonant frequencies, graphene, Monte Carlo simulation

## Abstract

With the distinguished properties in electronics, thermal conductivity, optical transparence and mechanics, graphene has a powerful potential in nanosensors, nano-resonators, supercapacitors, batteries, etc. The resonant frequency of graphene is an important factor in its application and working environment. However, the random dispersed porosities in graphene evidently change the lattice structure and destroy the integrity and geometrical periodicity. This paper focuses on the effects of random porosities in resonant frequencies of graphene. Monte Carlo simulation is applied to propagate the porosities in the finite element model of pristine graphene. The statistical results and probability density distribution of porous graphene with atomic vacancy defects are computed based on the Monte Carlo finite element model. The results of porous graphene with atomic vacancy defects are compared and discussed with the results of graphene with bond vacancy defects. The enhancement effects of atomic vacancy defects are confirmed in porous graphene. The influences of atomic vacancy defects on displacement and rotation vector sums of porous graphene are more concentrated in local places.

## 1. Introduction

Graphene is a two-dimensional (2D) nanomaterial composed of a hexagonal honeycomb lattice [1]. With the distinguished properties in electronics, thermal conductivity, optical transparence and mechanics, graphene has powerful potential in nanosensors, nano-resonators, supercapacitors [2,3,4], batteries [5,6,7], etc. The covalent bonds between carbon atoms in graphene ensure the stability in mechanical and chemical properties [8,9,10,11]. However, random porosities are an inevitable and significant issue in research and applications of graphene. On the one hand, the atomic [12,13,14] and bond [15,16] vacancy defects appear in the production process of graphene [17,18,19]. The effects of random porosities in graphene are important problems that must be confronted. On the other hand, the porosity in graphene not only leads to negative factors in the service environment, but also can be used and designed to enhance the competence in hydrogen storage and release [20,21,22] the piezoelectric effects after polarization [23,24] and other positive influences in the applications.

The challenges confronted for the study of random porosities in graphene are mainly concentrated around three aspects. First, the small size on the nanometer scale makes the precise measurement in physical experiments difficult and inconvenient, and the experimental equipment is supposed to satisfy more advanced and strict requirements [25,26,27]. Second, the random distributed porosities in graphene contribute to the deviation and variances in the results no matter which are measured from experiments or computed in the numerical simulations [28,29]. The confusion in uncertain results of porous graphene sets up obstacles for the comprehensive understanding of the graphene’s properties [30]. Third, the general concerns about an independent parameter ignore the correlation and relationships between parameters corresponding to mechanical and physical properties [31]. For example, resonant frequencies are related with both mass and stiffness of porous graphene [32]. Therefore, this study is aimed at analyzing the effects of random porosities in the resonant frequencies of graphene.

In the investigation of porous graphene, the experimental, theoretical and numerical methods are the effective ways of knowledge exploration. Using an atomic force microscope, nano-indention is performed and detected in the center of a suspended monolayer graphene membrane [19]. Besides, the Raman spectrum is a useful supplement in the experimental measurements of graphene [33]. In addition, tight-binding potentials [34,35,36], density function theory (DFT) [37,38,39] and molecular dynamics (MD) simulation [40,41] are the frequently used approaches. Gupta [42] used MD simulation and the predicted resonant frequencies were 1.7581 THz, 4.0706 THz, 4.7201 THz and 7.0325 THz in the first- to fourth-order vibration modes, respectively. By MD simulation, Khatibi [43] obtained 1.6030 THz, 2.4970 THz, 2.5980 THz and 3.5770 THz for pristine graphene. Furthermore, based on the DFT, Kudin [44], Liu [45] and Wei [46] provided approximate resonant frequencies for graphene. Additionally, Cadelano [47], Zhou [48] and Reddy [49] also have done related work in the vibration analysis of graphene. Chu [50] proposed the Monte Carlo simulation (MCS) to propagate random porosities in pristine graphene for the computation of resonant frequencies.

In order to take random porosities into consideration, atomic vacancy defects are dispersed in graphene by MCS. The stochastic sampling process in MCS provides sufficient random numbers, which correspond to the serial numbers of atoms in graphene. The marked atoms in the MCS form atomic vacancy defects with three connected bonds. The results of porous graphene are compared with the previous work of random bond vacancy defects in graphene. The analysis of porous graphene in vibration is useful and helpful to understand the mechanical properties of graphene in the real service environment. The random distributed porosities are among the most important factors that contribute to uncertainties in graphene. It is necessary to have quantitative computation and effective propagation for porosities in the reliability and stability analysis of graphene.

This article is structured as follows: In Section 2, the random atomic vacancy defects are introduced in pristine graphene, and the finite element model for porous graphene is created based on the continuum theory. Monte Carlo simulation is used to propagate the random distributed atomic vacancy defects in graphene. In Section 3, based on mathematical statistics and probability analysis, resonant frequencies of porous graphene are compared with the reported results in literature [35,36,37,38,39,40,41,42,43]. Furthermore, Section 3 also provides the discussion about the effects of atomic vacancy defects and bond-breaking defects in the vibration behavior of porous graphene. The last section offers a brief summary of this paper.

## 2. Results and Discussion

### 2.1. Statistical Results

Given that the porosities are randomly dispersed in graphene, sufficient times of performing the Monte Carlo-based stochastic finite element method (MC-SFEM) are necessary to simulate the uncertainties in the location of porosities. In this study, the repetition time of MC-SFEM is settled as 1000 for porous graphene. The database of the stochastic finite element model for porous graphene is huge. The statistical results of resonant frequencies are computed from the original database of MC-SFEM. The mean, maximum, minimum and variance values of resonant frequencies of porous graphene with the corresponding percentage of atomic vacancy defects are listed in Table 1.

**Figure 1:**
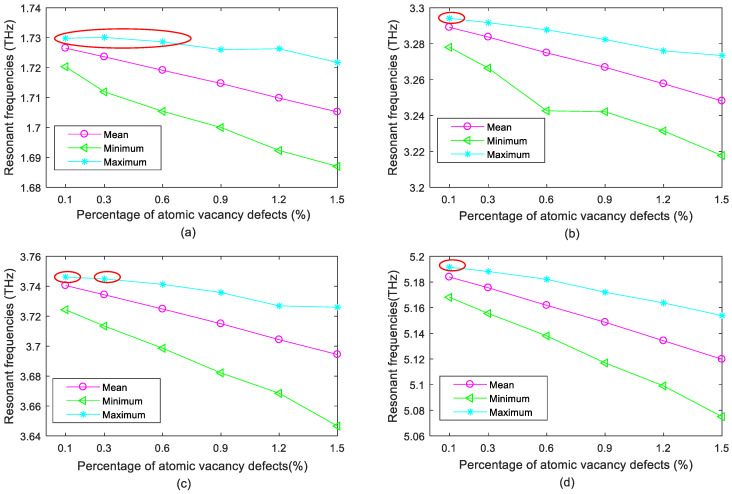
Statistical results of resonant frequencies for porous graphene ((**a**–**d**) are for the first- to fourth-order vibration modes, respectively; the red ellipses mark the points that larger than the corresponding value of the initial grapehene).

The mean values of resonant frequencies in porous graphene are computed from all the results in the sampling space. The maximum and minimum values of resonant frequencies in different vibration modes are tracked and captured in the result sets. The maximum and minimum values of resonant frequencies represent the extreme situations that can possibly appear in porous graphene. Besides the mean values of resonant frequencies in the statistical results, the maximum and minimum values also provide meaningful information as demonstrated in Figure 1.

With the increase of the amount of atom vacancy defects in porous graphene, the mean values of resonant frequencies linearly decrease in the first- to fourth-order vibration modes. However, the minimum and maximum values of resonant frequencies are more complicated with fluctuations. More importantly, the enhancement effects by atomic vacancy defects are observed in Figure 1 (marked with red ellipses). Even though the mean and minimum values of resonant frequencies in the first- to fourth-order vibration modes are smaller than those of pristine graphene, the maximum values of resonant frequencies illustrate the possibility of improving the resonant frequencies by atomic vacancy defects in porous graphene.

In Figure 1, when the percentage of atomic vacancy defects equals 0.1%, the maximum resonant frequencies in the first- to fourth-order vibration modes all exceed those of pristine graphene. In addition, the maximum resonant frequency of the first-order vibration mode is higher than that of pristine graphene when Per is smaller than 0.6%. Furthermore, the enhancement effects of atomic vacancy defects in porous graphene also happen when Per is 0.3% in the third-order vibration mode as shown in Figure 1c. Therefore, the introduction of appropriate atomic vacancy defects in pristine graphene contributes to the improvement of resonant frequencies in vibration modes.

Resonant frequencies are the quotient of stiffness and mass matrices of porous graphene. The atomic vacancy defects in porous graphene cause the reduction of mass, which is the numerator in the computation of resonant frequencies. The atomic vacancy defects in porous graphene also play roles in the weakening effects in stiffness matrices, which are the denominators. When weakening effects in stiffness matrices are smaller than the reduction in mass matrices, the resonant frequencies are amplified. By contrast, when the decrease of stiffness matrices of graphene is more dominant than that of mass matrices, the resonant frequencies are cut down. Therefore, the enhancement effects happening in minor situations with the tiny percentage of atomic vacancy defects are reasonable.

**Figure 2:**
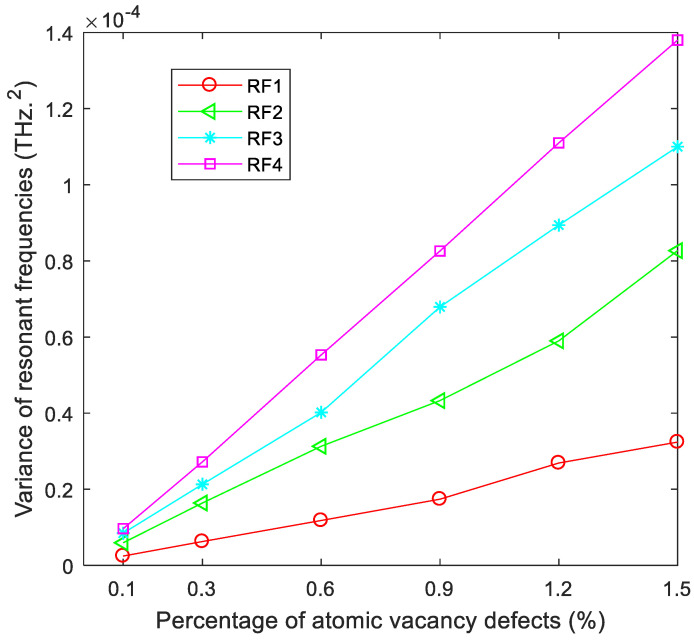
Variance of resonant frequencies for porous graphene (RF1-RF4 are the variances of resonant frequencies in the first- to fourth-order vibration modes, respectively).

In addition, Figure 2 presents the variance of resonant frequencies in different vibration modes. In each vibration mode, the variance of resonant frequencies becomes larger with the increase of atomic vacancy defects. Besides, when porous graphene has the same amount of atomic vacancy defects, the variance in low-order vibration modes is smaller than that of high-order vibration modes. For example, the variance of porous graphene with 0.3% of atomic vacancy defects in the first-order vibration mode is smaller than that in the second-, third- and fourth-order vibration modes. Furthermore, the gradient of the variance of resonant frequencies in high-order vibration modes is larger than that of low-order vibration modes. This means that with the increase of the amount of atomic vacancy defects, the amplification of the variance of resonant frequencies is faster in high-order vibration modes than in low-order ones. Thus, porous graphene has a stronger capacity to reduce the fluctuation and deviation in low-order vibration modes than in high-order vibration modes.

**Figure 3:**
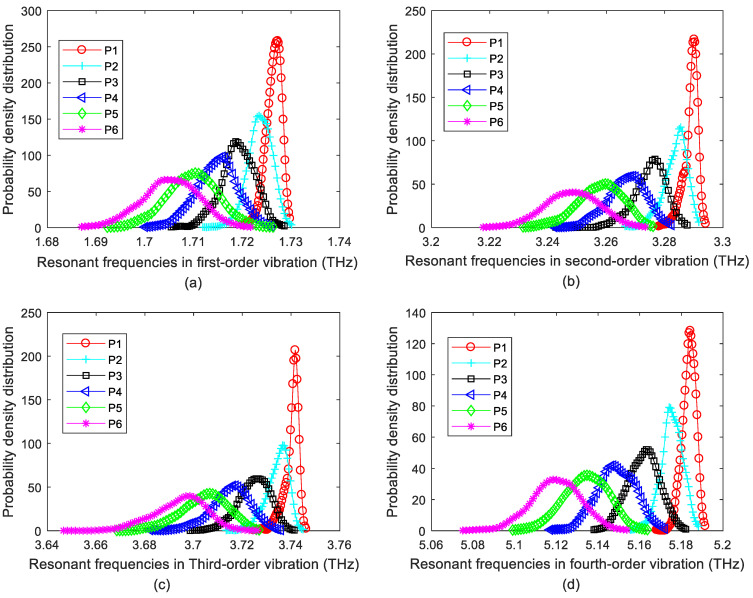
Probability density distribution of resonant frequencies for porous graphene (P1–P6 are for the 0.1%, 0.3%, 0.6%, 0.9%, 1.2% and 1.5% of atomic vacancy defects, respectively; (**a**–**d**) are for the first-order, second-order, third-order and fourth-order resonant vibration, respectively).

The probability density distribution of resonant frequencies in porous graphene is illustrated in Figure 3. The results in Figure 3 have a good agreement with those in Figure 1 and Figure 2. First, when the amount of atomic vacancy defects is 0.1%, the probability density of resonant frequencies is distributed in the narrower interval range than that of 0.3%, 0.6%, 0.9%, 1.2% and 1.5% in the first four vibration modes. This point confirms that the variance of resonant frequencies increases with the augmentation of atomic vacancy defects. Second, the peak of probability density moves to the left with the increase of the amount of atomic vacancy defects, which proves that the mean value of resonant frequencies reduces with the increase of atomic vacancy defects. Third, the probability density distribution of resonant frequencies in porous graphene is approximated to the shape of the Gaussian and Weibull density distribution, but the precise results of MC-SFEM are not as regular as those of the Gaussian or Weibull density distribution.

### 2.2. Comparison and Discussion

Atomic vacancy defects are formed by the disappearance of atoms with three connected neighbor bonds, while bond vacancy defects are the absence of single bonds. In the honeycomb lattice of graphene, one atom is connected with three neighbor bonds, and one bond is the link between two atoms. In order to compare the atomic and bond vacancy defects in graphene, the statistical results of resonant frequencies for porous graphene with bond vacancy defects are listed in Table 2.

**Figure 4:**
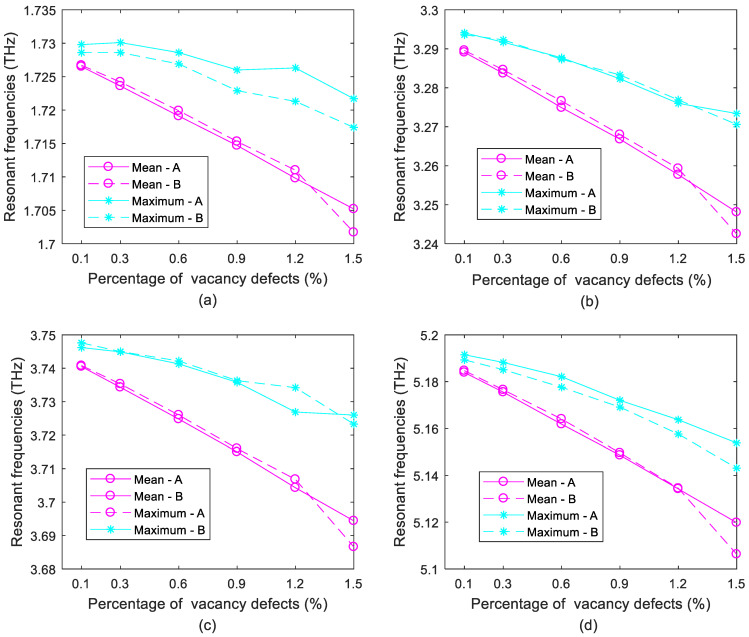
Comparison of atomic and bond vacancy defects (**a**–**d**) for the first- to fourth-order vibration modes; A and B represent the atomic and bond vacancy defects, respectively.

In Figure 4, the mean and maximum values of resonant frequencies in porous graphene are compared. On the one hand, the mean values of porous graphene with bond vacancy defects are close to those with atomic vacancy defects in different vibration modes. When *Per_b_* equals *Per,* the mean values of resonant frequencies in porous graphene with bond vacancy defects are a little larger than those with atomic vacancy defects, except when *Per_b_* is 1.5%; then, the mean values of resonant frequencies in porous graphene with bond vacancy defects are smaller than those with atomic vacancy defects. With the increment of bond vacancy defects in porous graphene, the mean values of resonant frequencies also decrease. However, the regularity of atomic vacancy defects enables porous graphene with a more solid ability to resist the reduction in resonant frequencies, especially when the amount of vacancy defects is large, such as 1.5%. On the other hand, when *Per_b_* is equivalent to *Per*, the maximum values of resonant frequencies in porous graphene with atomic vacancy defects are larger than those with bond vacancy defects. The contrary phenomena are rare. Enhancement effects in porous graphene with bond vacancy defects are also observed, but are smaller than those with atomic vacancy defects. Therefore, porous graphene with atomic vacancy defects not only has a more solid robustness in the reduction of resonant frequencies, but also can result in stronger possible enhancement effects.

**Figure 5:**
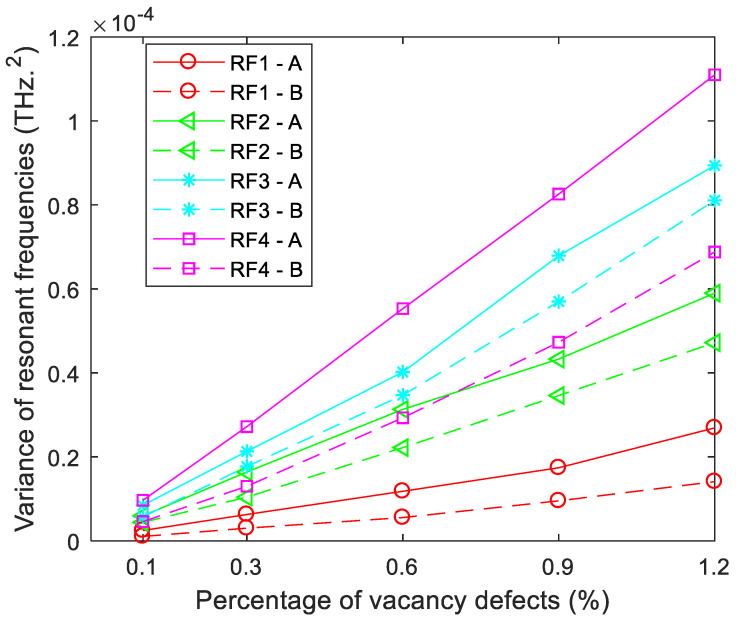
Variance of resonant frequencies for porous graphene (RF1-RF4 are the variance of resonant frequencies in the first- to fourth-order vibration modes; A and B represent the atomic and bond vacancy defects, respectively).

The variance values of resonant frequencies in porous graphene with two different vacancy defects are compared in Figure 5. The variance values of resonant frequencies in porous graphene with bond vacancy defects are evidently smaller than those with atomic vacancy defects. As mentioned above, each atom vacancy defect is connected with three neighbor bonds, and each bond links two atoms. In a sense, when *Per* equals *Per_b_*, the amount of bond vacancy defects in porous graphene with atomic vacancy defects is larger than that with bond vacancy defects and is approximately 1.5 times that with bond vacancy defects. Even though the regularity in atomic vacancy defects causes robustness in resonant frequencies and provides more evident enhancement effects in porous graphene, bond vacancy defects lead to smaller variance and deviation.

### 2.3. Vibration Modes of Porous Graphene

**Figure 6:**
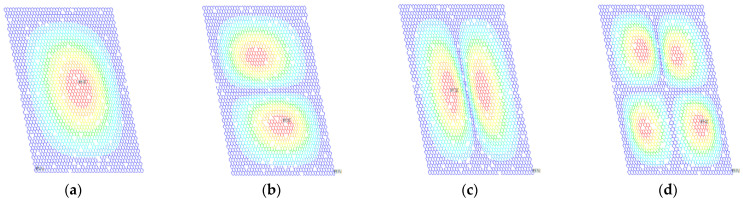
Displacement vector sums of porous graphene (**a**–**d**) for the first- to fourth-order vibration modes, respectively; the percentage of atomic vacancy defects is 1.5%.

**Figure 7:**
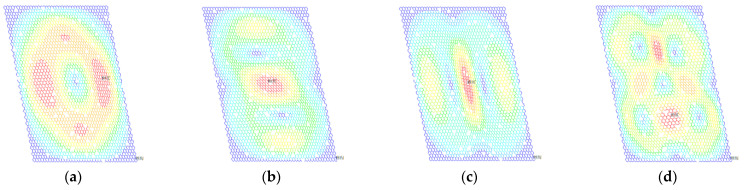
Rotation vector sums of porous graphene (**a**–**d**) for the first- to fourth-order vibration modes, respectively; the percentage of atomic vacancy defects is 1.5%.

The random distributed atomic vacancy defects cause the deviation of resonant frequencies in porous graphene. The vibration modes of one example in the MC-SFEM for porous graphene are depicted in Figure 6 and Figure 7.

Different from the influence of 5% bond vacancy defects in the literature [50], the geometrical symmetry and regularity in the vibration modes are not obviously destroyed in porous graphene with 1.5% atomic vacancy defects. However, atomic vacancy defects bring about changes in the local placement of graphene in the results of displacement and rotation vector sums. Vacancy defects caused by the absence of atoms are more concentrated than bond vacancy defects in graphene. Although atomic vacancy defects are stochastically distributed in porous graphene, the disappeared bonds are clustered around the absent atom. Therefore, atomic vacancy defects have more concentrated impacts on the results of displacement and rotation for the local scope.

## 3. Materials and Methods

### 3.1. Porous Graphene

The carbon atoms in graphene are combined with covalent bonds in sp^2^ hybrid orbitals. The carbon–carbon (C–C) covalent bonds are supposed to be the elastic beam elements in the characteristic lattice of graphene. The equivalent Young’s modulus and Poisson ratio are derived from the following equations.

The analytical function representing bond energy is the Morse function [51]:

(1) $$U_{r}=D_{ij}^{e}\left[e^{\left(-2a_{ij}\Delta r_{ij}\right)}-2e^{\left(-a_{ij}\Delta r_{ij}\right)}\right]$$

where $D_{ij}^{e}$ is the bond stretching energy, $r_{ij}$ is the equilibrium distance, $\Delta r_{ij}$ represents the variation of the bond length and $a_{ij}$ is a relative coefficient. With the parameter for hybridized sp^2^ bonds, the Morse potential is expressed as:

(2) $$U_{r}=D_{e}\left\{{\left[1-e^{-\beta (r-r_{0})}\right]}^{2}-1\right\}$$

where $r_{0}$ is the bond equilibrium length, $D_{e}$ is the energy of dissociation and β is the coefficient of regression fitting.

(3) $$r_{0}=0.139\;nm,\;D_{e}=6.03105\times 10^{-10}\;Nnm,\;\beta =26.25\;nm^{-1}$$

The energy of the bond angle is written as follows:

(4) $$U_{\theta }=\frac{1}{2}k_{\theta }{\left(\Delta \theta \right)}^{2}\left[1+k_{sextic}{\left(\Delta \theta \right)}^{4}\right]$$

with $k_{\theta }=0.9\times 10^{-18}\;Nm/rad^{2},\;\Delta \theta =\theta -\theta_{0},\;\theta_{0}=2.094\;rad,\;k_{sextic}=0.754\;rad^{-4}$.

Therefore, the equivalent parameters, the diameter d, Young’s modulus E and shear modulus G of the beam elements representing the C–C bonds can be computed as follows:

(5) $$\left\{ \begin{array}{l} d=4\sqrt{\frac{k_{\theta }}{k_{r}}}\\ E=\frac{k_{r}^{2}L}{4\pi k_{\theta }}\\ G=\frac{k_{r}^{2}k_{\tau }L}{8\pi k_{\theta }^{2}} \end{array}\right.$$

where $k_{r},\;k_{\theta },\;k_{\tau }$ are the bond sketching, bond bending and torsional resistance force constants, respectively. Thus, the related parameters in small scale for graphene are transformed into the material parameters, which can be directly used in the finite element model for mechanical analysis.

The C–C bonds are simplified as the beam finite element model in pristine graphene. The beam finite element has a circular solid cross-sectional area. The length of each beam in the finite element model corresponds to the distance between the neighboring atoms in the honeycomb lattice of graphene. The original finite element model for graphene without vacancy defects was verified in our previous work, which reached a good agreement with the results computed by molecular dynamics, density function theory, etc. [50]. Compared with the adaptive intermolecular reactive empirical bond order (AIREBO) potential method in molecular dynamics for C–C bonds, the finite element model for graphene not only has merits in computational costs, but also is more competitive in the macro-property analysis for graphene since the resonant frequency in this study is an intrinsic macro-characteristic.

### 3.2. Beam Finite Element

The beam finite element used in this study is based on Timoshenko beam theory which includes first-order shear deformation effects. The element is a linear, quadratic and cubic two-node beam element in 3D. For each node, it has six degrees of freedom, which include translations in the x, y and z directions and rotations around the x-, y- and z-axes. The beam finite element is well-suited for linear, large rotation and/or large-strain nonlinear applications. Different from the truss element, the beam finite element has the capacities in axial and flexural computation.

The equation derived by Timoshenko that governs flexural vibrations of beams with a constant cross-section can be expressed as follows [50]:

(6) $$\frac{EI}{\rho A}\frac{\partial^{4}\xi }{\partial z^{4}}-\frac{I}{A}(1+\frac{E}{\kappa G})\frac{\partial^{4}\xi }{\partial z^{2}\partial t^{2}}+\frac{\partial^{2}\xi }{\partial t^{2}}+\frac{\rho I}{\kappa GA}\frac{\partial^{4}\xi }{\partial z^{4}}=0$$

where ξ=ξ(z,t) is the transversal displacement along the x-axis at point *z* and time *t*, *E* is the Young’s modulus, I is the inertia moment, *G* is the shear modulus, ρ is the mass density and *A* is the cross-section area. In this theory, Timoshenko shear coefficient κ is a free parameter.

Besides ξ, angular variable θ is introduced. During a flexural motion, cross-sections are supposed to remain flat and perpendicular to the deflected neutral axis at any point of this axis. Angle θ between the z-axis and the vector orthogonal to the cross-section is equal to the angle between the neutral axis tangent line and the z-axis. Note that θ equals the slope of the deflected neutral axis, that is,

(7) $$\theta \approx \tan\theta =\frac{\partial \xi }{\partial z}$$

In the normal mode, ξ(z,t) varies harmonically with time as follows:

(8) ξ(z,t)=[Acos(wt)+Bsin(wt)]χ(z)=Csin(wt+φ)χ(z)

where *A*, *B*, *C* and φ are the corresponding constants to be determined. Equation w=2πf is the angular frequency and χ(z) is a function that determines the normal mode amplitude.

(9) $$\frac{\partial^{4}\chi }{\partial z^{4}}+\frac{\rho w^{2}}{M_{r}}\frac{\partial^{2}\chi }{\partial z^{2}}+\frac{w^{2}\rho^{2}}{\kappa GE}[w^{2}-w_{c}^{2}]\chi =0$$

with $w_{c}=2\pi f=\sqrt{\frac{\kappa GA}{\rho I}}$, where $f_{c}$ is the critical frequency and $\frac{1}{M_{r}}=\left(\frac{1}{E}+\frac{1}{\kappa G}\right)$ is the reduced modulus.

It is well known that solutions of the equation above behave differently according to $w^{2}-w_{c}^{2}$. The general solution can be written as follows:

(10) $$\left\{ \begin{array}{l} \chi (z)=A_{1}\sin(K_{1}z)+B_{1}\cos(K_{1}z)+C_{1}e^{K_{2}z}+D_{1}e^{-K_{2}z}\;w<w_{c}\;\\ \chi (z)=A_{2}\sin(K_{1}z)+B_{2}\cos(K_{1}z)+C_{2}\sin(K_{2}z)+D_{2}\cos(K_{2}z)\;w>w_{c} \end{array}\right.$$

where

(11) $$\left\{ \begin{array}{l} K_{1}=\sqrt{\frac{\rho w^{2}}{2M_{r}}+\sqrt{{\left(\frac{\rho w^{2}}{2M_{r}}\right)}^{2}-\frac{\rho^{2}w^{2}}{\kappa GE}(w^{2}-w_{c}^{2})}}\\ K_{2}=\sqrt{S\left[\frac{\rho w^{2}}{2M_{r}}-\sqrt{{\left(\frac{\rho w^{2}}{2M_{r}}\right)}^{2}-\frac{\rho^{2}w^{2}}{\kappa GE}(w^{2}-w_{c}^{2})}\right]} \end{array}\right.\;With\;S=\left\{ \begin{array}{l} 1\;if\;w>w_{c}\;\\ -1\;if\;w<w_{c}\; \end{array}\right.$$

Usually, coefficients $A_{i},\;B_{i},\;C_{i},\;D_{i}$ are different from zero, and the solutions of equations include functions depending on both $K_{1}$ and $K_{2}$, where $K_{1}$ and $K_{2}$ are defined as positive square roots.

For free vibration analysis for Timoshenko beam based on the principle of virtual work, the weak form of equation can be written as follows:

(12) $$\int_{0}^{L}EI\frac{\partial \theta }{\partial x}\delta (\frac{\partial \theta }{\partial x})dx+\int_{0}^{L}\kappa GA(\frac{\partial \xi }{\partial x}-\theta )\delta (\frac{\partial \xi }{\partial x}-\theta )dx=\int_{0}^{L}\delta \xi \rho A\ddot{\xi }dx+\int_{0}^{L}\delta \theta \rho I\ddot{\theta }dx$$

As defined above, ξ is the transversal displacement in Timoshenko beam, where θ is the transversal rotation, while $\ddot{\xi }$ and $\ddot{\theta }$ are the transverse and rotary accelerations, respectively, *L* is the length of the beam and δ denotes that the terms are virtual.

### 3.3. Monte Carlo-Based Finite Element Method

MCS is a classical stochastic sampling method with a solid mathematical foundation [32]. Each atom in the honeycomb lattice of pristine graphene is marked with different serial numbers in the finite element model. MCS is used to provide the stochastic serial numbers that are related to the atoms forming vacancy defects. Once the specific atoms are selected in the stochastic sampling process of MCS, three connected C–C bonds disappear to develop into the atomic vacancy defects in the local location.

The finite element model of pristine graphene consists of 4212 atoms (*N_a_*), 6226 bonds (*N_b_*) and 18,678 elements. Therefore, the percentage of atomic vacancy defects in porous graphene can be defined as follows:

(13) $$Per=\frac{D_{a}}{N_{a}}$$

**Figure 8:**
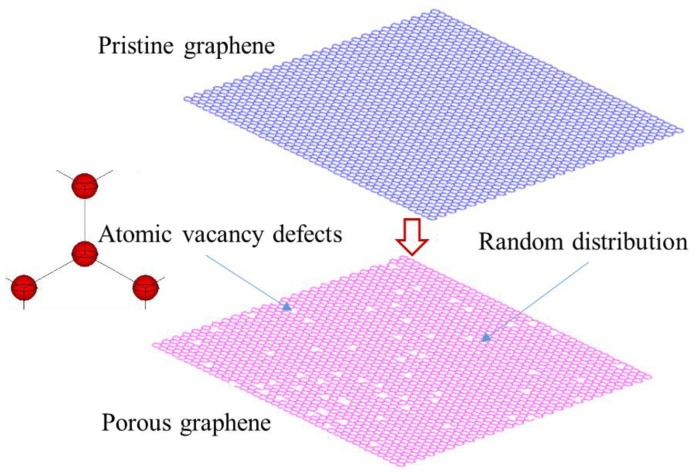
Schematic of porous graphene (the percentage of atomic vacancy defects is 1.5%).

Similarly, the percentage of bond vacancy defects is computed as follows:

(14) $$Per_{b}=\frac{D_{b}}{N_{b}}$$

where *D_a_* and *D_b_* are the amounts of atomic and bond vacancy defects, respectively. Figure 8 illustrates the schematic of porous graphene with 1.5% atomic vacancy defects in the finite element model.

In this paper, MCS is applied to propagate the porosities in the finite element model of pristine graphene as shown in Figure 8. By the combination of MCS with finite element computation, the random distributed atomic vacancy defects are stochastically introduced in the finite element model of graphene. The implementation of finite element computation provides resonant frequencies of graphene. Then, the impacts of atomic vacancy defects can be discussed and analyzed depending on the proposed MC-SFEM based on a huge database with a large sample space.

## 4. Conclusions

In this paper, random atomic vacancy defects are taken into consideration in the vibration analysis of porous graphene. The MC-SFEM is applied to propagate stochastic porosities in pristine graphene and compute resonant frequencies. Statistical results and probability density distribution for porous graphene with atomic vacancy defects are computed and carried out. Based on this work, the following key points can be emphasized:

- Probability density distributions of resonant frequencies caused by random distributed atomic vacancy defects are not as regular as the Gaussian or Weibull distribution.
- Resonant frequencies can be amplified by the introduction of appropriate atomic vacancy defects in pristine graphene.
- Porous graphene has a stronger capacity to reduce fluctuations and deviations in low-order vibration modes than in high-order vibration modes.
- The porosities in graphene not only ensures a more solid robustness in the reduction of resonant frequencies, but also can result in stronger possible enhancement effects.
- The impacts of atomic vacancy defects are more concentrated in the local scope.

## Figures and Tables

**Table 1:** Statistical results of resonant frequencies for porous graphene with atomic vacancy defects.

Per (%)	Mode	Mean (THz)	Minimum (THz)	Maximum (THz)	Variance
0.1	1	1.7265	1.7203	1.7298	2.46 × 10^−6^
	2	3.2891	3.2780	3.2941	5.95 × 10^−6^
	3	3.7405	3.7243	3.7462	8.47 × 10^−6^
	4	5.1839	5.1681	5.1915	9.64 × 10^−6^
0.3	1	1.7236	1.7119	1.7301	6.27 × 10^−6^
	2	3.2837	3.2664	3.2917	1.64 × 10^−5^
	3	3.7343	3.7134	3.7449	2.13 × 10^−5^
	4	5.1755	5.1555	5.1882	2.72 × 10^−5^
0.6	1	1.7191	1.7054	1.7286	1.18 × 10^−5^
	2	3.2749	3.2426	3.2877	3.13 × 10^−5^
	3	3.7248	3.6986	3.7413	4.02 × 10^−5^
	4	5.1618	5.1380	5.1821	5.53 × 10^−5^
0.9	1	1.7147	1.7000	1.7260	1.74 × 10^−5^
	2	3.2668	3.2421	3.2823	4.33 × 10^−5^
	3	3.7150	3.6821	3.7358	6.79 × 10^−5^
	4	5.1486	5.1169	5.1720	8.26 × 10^−5^
1.2	1	1.7098	1.6923	1.7263	2.69 × 10^−5^
	2	3.2577	3.2314	3.2760	5.90 × 10^−5^
	3	3.7043	3.6685	3.7269	8.94 × 10^−5^
	4	5.1342	5.0990	5.1637	1.11 × 10^−4^
1.5	1	1.7052	1.6870	1.7217	3.24 × 10^−5^
	2	3.2481	3.2177	3.2734	8.27 × 10^−5^
	3	3.6944	3.6466	3.7260	1.10 × 10^−4^
	4	5.1198	5.0750	5.1538	1.38 × 10^−4^

**Table 2:** Statistical results of resonant frequencies for porous graphene with bond vacancy defects.

Per (%)	Mode	Mean (THz)	Minimum (THz)	Maximum (THz)	Variance
0.1	1	1.7267	1.7220	1.7286	1.04 × 10^−6^
	2	3.2896	3.2783	3.2936	4.40 × 10^−6^
	3	3.7408	3.7304	3.7476	5.70 × 10^−6^
	4	5.1847	5.1766	5.1893	4.58 × 10^−6^
0.3	1	1.7242	1.7166	1.7286	3.01 × 10^−6^
	2	3.2846	3.2664	3.2923	1.04 × 10^−5^
	3	3.7353	3.7140	3.7450	1.77 × 10^−5^
	4	5.1765	5.1637	5.1851	1.30 × 10^−5^
0.6	1	1.7199	1.7108	1.7269	5.54 × 10^−6^
	2	3.2766	3.2579	3.2873	2.22 × 10^−5^
	3	3.7260	3.7010	3.7422	3.47 × 10^−5^
	4	5.1640	5.1323	5.1776	2.93 × 10^−5^
0.9	1	1.7153	1.7038	1.7229	9.52 × 10^−6^
	2	3.2680	3.2480	3.2833	3.46 × 10^−5^
	3	3.7160	3.6912	3.7363	5.70 × 10^−5^
	4	5.1496	5.1242	5.1691	4.73 × 10^−5^
1.2	1	1.7110	1.6962	1.7213	1.41 × 10^−5^
	2	3.2593	3.2323	3.2769	4.72 × 10^−5^
	3	3.7068	3.6723	3.7342	8.11 × 10^−5^
	4	5.1345	5.1003	5.1576	6.88 × 10^−5^
1.5	1	1.7017	0	1.7174	5.83 × 10^−3^
	2	3.2425	0	3.2706	2.12 × 10^−2^
	3	3.6866	0	3.7233	2.74 × 10^−2^
	4	5.1063	0	5.1431	5.24 × 10^−2^

## References

[1] Novoselov K.S., Geim A.K., Morozov S.V., Jiang D., Zhang Y., Dubonos S.V., Grigorieva I.V., Firsov A.A. (2004). Electric field effect in atomically thin carbon films. Science.

[2] Pruna A., Tamvakos D., Sgroi M., Pullini D., Nieto E.A., Busquets-Mataix D. (2015). Electrocapacitance of hybrid film based on graphene oxide reduced by ascorbic ac-id. Int. J. Mater. Res..

[3] Pullini D., Siong V., Tamvakos D., Ortega B.L., Sgroi M., Veca A., Glanz C., Kolaric I., Pruna A. (2015). Enhancing the capacitance and active surface utilization of supercapacitor electrode by graphene nanoplatelets. Compos. Sci. Technol..

[4] Ke Q., Wang J. (2016). Graphene-based materials for supercapacitor electrodes—A review. J. Mater..

[5] Cheng Q., Okamoto Y., Tamura N., Tsuji M., Maruyama S., Matsuo Y. (2017). Graphene-Like-Graphite as Fast-Chargeable and High-Capacity Anode Materials for Lithium Ion Batteries. Sci. Rep..

[6] Cai X., Lai L., Shen Z., Lin J. (2017). Graphene and graphene-based composites as Li-ion battery electrode materials and their appli-cation in full cells. J. Mater. Chem. A.

[7] Sgroi M.F., Pullini D., Pruna A.I. (2020). Lithium Polysulfide Interaction with Group III Atoms-Doped Graphene: A Computational Insight. Batteries.

[8] Bonilla L.L., Carpio A. (2011). Theory of defect dynamics in graphene: Defect groupings and their stability. Contin. Mech. Thermodyn..

[9] Kim H.S., Oweida T.J., Yingling Y.G. (2017). Interfacial stability of graphene-based surfaces in water and organic solvents. J. Mater. Sci..

[10] Ariza M.P., Ortiz M., Serrano R. (2010). Long-term dynamic stability of discrete dislocations in graphene at finite temperature. Int. J. Fract..

[11] Rani P., Jindal V.K. (2013). Stability and electronic properties of isomers of B/N co-doped graphene. Appl. Nanosci..

[12] Nayebi P., Zaminpayma E., Emami-Razavi M. (2018). Study of electronic properties of graphene device with vacancy cluster defects: A first principles approach. Thin Solid Films.

[13] Li T., Yarmoff J.A. (2018). Defect-induced oxygen adsorption on graphene films. Surf. Sci..

[14] Araujo E.N.D., Brant J.C., Archanjo B.S., Medeiros-Ribeiro G., Alves E.S. (2018). Quantum corrections to conductivity in graphene with vacancies. Phys. E Low Dimens. Syst. Nanostruct..

[15] Son J., Choi M., Choi H., Kim S.J., Kim S., Lee K.-R., Vantasin S., Tanabe I., Cha J., Ozaki Y. (2016). Structural evolution of graphene in air at the electrical breakdown limit. Carbon.

[16] Okada T., Inoue K.Y., Kalita G., Tanemura M., Matsue T., Meyyappan M., Samukawa S. (2016). Bonding state and defects of nitrogen-doped graphene in oxygen reduction reaction. Chem. Phys. Lett..

[17] Geim A.K. (2009). Graphene: Status and Prospects. Science.

[18] Grantab R., Shenoy V.B., Ruoff R.S. (2010). Anomalous Strength Characteristics of Tilt Grain Boundaries in Graphene. Science.

[19] Terdalkar S.S., Huang S., Yuan H., Rencis J.J., Zhu T., Zhang S. (2010). Nanoscale fracture in graphene. Chem. Phys. Lett..

[20] Tozzini V., Pellegrini V. (2011). Reversible Hydrogen Storage by Controlled Buckling of Graphene Layers. J. Phys. Chem. C.

[21] Roszak R., Firlej L., Roszak S., Pfeifer P., Kuchta B. (2016). Hydrogen storage by adsorption in porous materials: Is it possible?. Colloids Surf. A Physicochem. Eng. Asp..

[22] Yadav S., Zhu Z., Singh C.V. (2014). Defect engineering of graphene for effective hydrogen storage. Int. J. Hydrogen Energy.

[23] Hinchet R., Khan U., Falconi C., Kim S.-W. (2018). Piezoelectric properties in two-dimensional materials: Simulations and experiments. Mater. Today.

[24] Kundalwal S.I., Meguid S.A., Weng G.J. (2017). Strain gradient polarization in graphene. Carbon.

[25] Lee C., Wei X., Kysar J.W., Hone J. (2008). Measurement of the elastic properties and intrinsic strength of monolayer graphene. Science.

[26] Eckmann A., Felten A., Mishchenko A., Britnell L., Krupke R., Novoselov K.S., Casiraghi C. (2012). Probing the Nature of Defects in Graphene by Raman Spectroscopy. Nano Lett..

[27] Shi J., Chu L., Braun R. (2019). A kriging surrogate model for uncertainty analysis of graphene based on a finite element method. Int. J. Mol. Sci..

[28] Qin H., Sun Y., Liu J.Z., Liu Y. (2016). Mechanical properties of wrinkled graphene generated by topological defects. Carbon.

[29] Chu L., Shi J., Ben S. (2018). Buckling Analysis of Vacancy-Defected Graphene Sheets by the Stochastic Finite Element Method. Materials.

[30] Deng S., Berry V. (2016). Wrinkled, rippled and crumpled graphene: An overview of formation mechanism, electronic properties, and applications. Mater. Today.

[31] Zandiatashbar A., Lee G.-H., An S.J., Lee S., Mathew N., Terrones M., Hayashi T., Picu C.R., Hone J., Koratkar N. (2014). Effect of defects on the intrinsic strength and stiffness of graphene. Nat. Commun..

[32] Chu L., Shi J., De Cursi E.S., Xu X., Qin Y., Xiang H. (2018). Monte Carlo-Based Finite Element Method for the Study of Randomly Distributed Vacancy Defects in Graphene Sheets. J. Nanomater..

[33] Ferrari A.C., Meyer J.C., Scardaci V., Casiraghi C., Lazzeri M., Mauri F., Piscanec S., Jiang D., Novoselov K.S., Roth S. (2006). Raman Spectrum of Graphene and Graphene Layers. Phys. Rev. Lett..

[34] Mendez J.P., Ariza M.P. (2016). Harmonic model of graphene based on a tight binding interatomic potential. J. Mech. Phys. Solids.

[35] De Oliveira Neto P.H., Van Voorhis T. (2018). Dynamics of charge quasiparticles generation in armchair graphene nanoribbons. Carbon.

[36] Sinitsa A.S., Lebedeva I.V., Popov A.M., Knizhnik A.A. (2018). Long triple carbon chains formation by heat treatment of graphene nanoribbon: Mo-lecular dynamics study with revised Brenner potential. Carbon.

[37] Özkaya S., Blaisten-Barojas E. (2018). Polypyrrole on graphene: A density functional theory study. Surf. Sci..

[38] Maschio L., Lorenz M., Pullini D., Sgroi M., Civalleri B. (2016). The unique Raman fingerprint of boron nitride substitution patterns in gra-phene. Phys. Chem. Chem. Phys..

[39] Ganji M.D., Sharifi N., Ahangari M.G. (2014). Adsorption of H2S molecules on non-carbonic and decorated carbonic graphenes: A van der Waals density functional study. Comput. Mater. Sci..

[40] Tsai J.L., Tu J.F. (2010). Characterizing mechanical properties of graphite using molecular dynamics simulation. Mater. Des..

[41] Javvaji B., Budarapu P., Sutrakar V., Mahapatra D.R., Paggi M., Zi G., Rabczuk T. (2016). Mechanical properties of Graphene: Molecular dynamics simulations correlated to continuum based scaling laws. Comput. Mater. Sci..

[42] Gupta S., Dharamvir K., Jindal V.K. (2005). Elastic moduli of single-walled carbon nanotubes and their ropes. Phys. Rev. B.

[43] Sadeghzadeh S., Khatibi M.M. (2017). Modal identification of single layer graphene nano sheets from ambient re-sponses using frequency domain decomposition. Eur. J. Mech. A/Solids.

[44] Kudin K.N., Scuseria G.E., Yakobson B.I. (2001). C_2_F, BN, and C nanoshell elasticity from ab initio, computations. Phys. Rev. B.

[45] Liu F., Ming P., Li J. (2007). Ab initio, calculation of ideal strength and phonon instability of graphene under tension. Phys. Rev. B.

[46] Wei X., Fragneaud B., Marianetti C.A., Kysar J.W. (2009). Nonlinear elastic behavior of graphene: Ab initio calculations to con-tinuum description. Phys. Rev. B.

[47] Cadelano E., Palla P.L., Giordano S., Colombo L. (2009). Nonlinear Elasticity of Monolayer Graphene. Phys. Rev. Lett..

[48] Zhou L., Wang Y., Cao G. (2013). Elastic properties of monolayer graphene with different chiralities. J. Phys. Condens. Matter.

[49] Reddy C.D., Rajendran S., Liew K.M. (2006). Equilibrium configuration and continuum elastic properties of finite sized graphene. Nanotechnology.

[50] Chu L., Shi J., Souza de Cursi E. (2018). Vibration Analysis of Vacancy Defected Graphene Sheets by Monte Carlo Based Finite Element Method. Nanomaterials.

[51] Belytschko T., Xiao S.P., Schatz G.C., Ruoff R.S. (2002). Atomistic simulations of nanotube fracture. Phys. Rev. B.

